# Production, Purification and Characterization of Recombinant Biotinylated Phytochrome B for Extracellular Optogenetics

**DOI:** 10.21769/BioProtoc.3541

**Published:** 2020-03-05

**Authors:** Maximilian Hörner, O. Sascha Yousefi, Wolfgang W. A. Schamel, Wilfried Weber

**Affiliations:** 1 Faculty of Biology, University of Freiburg, Freiburg, Germany; 2 Signalling Research Centres BIOSS and CIBSS, University of Freiburg, Freiburg, Germany; 3 Center for Chronic Immunodeficiency (CCI), Medical Center Freiburg and Faculty of Medicine, University of Freiburg, Freiburg, Germany

**Keywords:** Optogenetics, Phytochrome B, PhyB, PhyB-AviTag, *E. coli*, Biotin, Protein, Extracellular

## Abstract

In the field of extracellular optogenetics, photoreceptors are applied outside of cells to obtain systems with a desired functionality. Among the diverse applied photoreceptors, phytochromes are the only ones that can be actively and reversibly switched between the active and inactive photostate by the illumination with cell-compatible red and far-red light. In this protocol, we describe the production of a biotinylated variant of the photosensory domain of *A. thaliana* phytochrome B (PhyB-AviTag) in *E. coli* with a single, optimized expression plasmid. We give detailed instructions for the purification of the protein by immobilized metal affinity chromatography and the characterization of the protein in terms of purity, biotinylation, spectral photoswitching and the light-dependent interaction with its interaction partner PIF6. In comparison to previous studies applying PhyB-AviTag, the optimized expression plasmid used in this protocol simplifies the production process and shows an increased yield and purity.

## Background

In the emerging field of extracellular optogenetics, photoreceptors are applied outside of cells, *e.g.*, to obtain control of biological function or to engineer light-responsive biomaterials (Leung *et al.*, 2008; Zhang *et al.*, 2015; Chen and Wegner, 2017; Lyu *et al.*, 2017; Wang *et al.*, 2017; Bartelt *et al.*, 2018; Beyer *et al.*, 2018a; Beyer *et al.*, 2018b; Jia *et al.*, 2018; Kolar *et al.*, 2018; Liu *et al.*, 2018; Wu *et al.*, 2018; Baaske *et al.*, 2019; Gil *et al.*, 2019; Hörner *et al.*, 2019a and 2019c; Sentürk *et al.*, 2019 and 2020; Yousefi *et al.*, 2019). Among the diverse used photoreceptors, phytochromes have the unique property that they can be actively and reversibly switched between the active and inactive photostate by the illumination with cell-compatible red and far-red light. Illumination with red 660 nm light shifts the equilibrium of the frequently applied photosensory domain of phytochrome B (PhyB, amino acids 1-651) from *A. thaliana* towards the Pfr (far-red light absorbing) state, in which the protein interacts with the phytochrome interacting factor 6 (PIF6) from *A. thaliana*. For this interaction, the first 100 amino acids of PIF6 are sufficient. Illumination with far-red 740 nm light switches PhyB into the Pr (red light absorbing) state and prevents interaction with PIF6. To couple PhyB to other proteins or biomaterials, we fused an AviTag biotinylation motif to PhyB (PhyB-AviTag). During protein production, the endogenous *E. coli* BirA ligase covalently couples biotin to the AviTag motif (Avidity) (Beckett *et al.*, 1999). In previous studies, we and others applied PhyB-AviTag encoded by the plasmid pMH17 (Table 1) (Smith *et al.*, 2016). Coexpression with the enzyme p171 (Essen *et al.*, 2008) encoding the biosynthesis enzymes HO1 and PcyA for the chromophore phycocyanobilin (PCB) resulted in the formation of functional PhyB-AviTag. In this protocol, we describe the production, purification and analysis of PhyB-AviTag encoded on the optimized expression plasmid pMH1105 (Table S1). This plasmid is based on our previously published plasmid pMH610 and encodes a codon-optimized variant of PhyB-AviTag as well as the biosynthesis enzymes heme oxygenase 1 (HO1) and PCB:ferredoxin oxidoreductase (PcyA), thus avoiding the need of co-transformation of two plasmids (Hörner *et al.*, 2019b). Moreover, compared with the previous production system, plasmid pMH1105 only requires IPTG for expression induction (no additional arabinose) and is compatible with high-cell-density *E. coli* fermentation. More importantly, it shows an increased yield and purity of the PhyB-AviTag, but maintains the same amino acid sequence of the established PhyB-AviTag encoded by plasmid pMH17. In this protocol, we show the procedure that was used to purify PhyB-AviTag for the light-controlled activation of the T cell receptor (Yousefi *et al.*, 2019) in detail. Additionally, we give comprehensive instructions for the characterization of PhyB-AviTag in terms of purity, biotinylation, spectral photoswitching and light-dependent interaction with PIF6. In the accompanying protocol, the production of PhyB-AviTag tetramers and their application as ligand for a PIF6-functionalized T cell receptor is described by Yousefi *et al.*, 2020.

**Table 1.** Overview of studies that applied PhyB-AviTag

Aim of study	PhyB-AviTag coupled via biotin to	Light-dependent PhyB-AviTag interaction partners	Reference
Activation of T cell receptor	DyLight650-conjugated streptavidin	GFP-PIF^S^-TCR	(Yousefi *et al.*, 2019)
Control of integrin-matrix interaction	NeutrAvidin-functionalized glass surface	Integrin αV-β3-PIF^S^ (OptoIntegrin)	(Baaske *et al.*, 2019)
Purification of protein complexes	NeutrAvidin-functionalized crosslinked agarose	GFP-PIF6 ZAP70-PIF6	(Hörner *et al.*, 2019a)
Trapping and release of proteins	Streptavidin-functionalized crosslinked agarose Avidin-functionalized glass surface NeutrAvidin-functionalized fibrin hydrogel	GFP-PIF6 ZZ-PIF6 TEV-mCherry-PIF6	(Beyer *et al.*, 2018b)
Counting biomaterial	Streptavidin-functionalized crosslinked agarose	TEV-mCherry-PIF6 GFP-PIF6 CrtI-PIF6 MBP-CrtY-GFP-PIF6	(Beyer *et al.*, 2018a)
Cargo attachment and release in bacteria-driven microswimmers	Streptavidin on *E. coli* surface	PIF6-GFP	(Sentürk *et al.*, 2020)

## Materials and Reagents

Note: If not stated otherwise, store all materials and reagents at room temperature. Use ultrapure water obtained by reverse osmosis for all solutions.

- Production of PhyB-AviTag in *E. coli*
  1. 50 ml conical centrifuge tubes (Corning, catalog number: 352070)
  2. 0.22 µm PES syringe filters (Carl Roth, catalog number: P668.1)
  3. BL21 Star (DE3) One Shot chemically competent *E. coli* (Thermo Fisher Scientific, catalog number: C601003), store at -80 °C
  4. Plasmid pMH1105 (available via Addgene, plasmid ID 131864. Addgene ships plasmids as transformed *E. coli* in a stab culture format. Purify plasmid from *E. coli* with a miniprep kit, *e.g.*, Wizard Plus SV Miniprep Kit, Promega, catalog number: A1330), store purified plasmid DNA at -20 °C. For plasmid sequence, see Table S1.
  5. Ice
  6. Cuvettes for photometer (VWR, catalog number: 634-0676)
  7. Liquid nitrogen
  8. Lysogeny broth (LB) (Luria/Miller, Carl Roth, catalog number: X968.1)
  9. Streptomycin sulfate (PanReac AppliChem, catalog number: A1852)
  10. Isopropyl β-D-1-thiogalactopyranoside (IPTG, Carl Roth, catalog number: CN08.4)
  11. Dulbecco’s phosphate buffered saline (PBS, Sigma-Aldrich, catalog number: D8537)
  12. D(+)-biotin (Carl Roth, catalog number: 3822.1)
  13. NaHCO_3_ (Sigma-Aldrich, catalog number: 31437-M)
  14. Tris(2-carboxyethyl)phosphine (TCEP) hydrochloride (Sigma-Aldrich, catalog number: C4706)
  15. N-2-Hydroxyethyl piperazine-N'-2-ethane sulphonic acid (HEPES, Carl Roth, catalog number: 9105.4)
  16. NaCl (Carl Roth, catalog number: 9265.2)
  17. Imidazole (Carl Roth, catalog number: X998.3)
  18. Glycerol (Carl Roth, catalog number: 3783.2)
  19. LB medium (see Recipes)
  20. 50 mg/ml streptomycin stock solution (see Recipes)
  21. 1 M IPTG stock solution (see Recipes)
  22. 5 mM biotin stock solution (see Recipes)
  23. 100 mM TCEP stock solution (see Recipes)
  24. Lysis buffer (see Recipes)
- Purification of PhyB-AviTag
  1. 1.5 ml microcentrifuge tubes (Sarstedt, catalog number: 72.690.001)
  2. Ice
  3. Ni-NTA Superflow column (Qiagen, catalog number: 30761), store at 4 °C
  4. Spin-X UF 20 centrifugal concentrators, 10 kDa MWCO, PES (Corning, catalog number: 431488)
  5. HiPrep 26/10 desalting column (GE Healthcare, catalog number: 17508701), store at 4 °C
  6. Bradford assay reagent (Bio-Rad, catalog number: 5000006), store at 4 °C
  7. Glycerol (Carl Roth, catalog number: 3783.2)
  8. Liquid nitrogen
  9. Bovine serum albumin (BSA, Sigma Aldrich, catalog number: A6003-25G), store at 4 °C
  10. Lysis buffer (see Recipes)
  11. Elution buffer (see Recipes)
  12. Protein buffer (see Recipes)
  13. 1 mg/ml BSA protein standard (see Recipes)
- Analysis of purified PhyB-AviTag
  1. 1.5 ml microcentrifuge tubes (Sarstedt, catalog number: 72.690.001)
  2. Black 96-well plates (Corning, catalog number: 3915)
  3. UV-transparent 96-well plates (Greiner Bio-One, catalog number: 655801)
  4. Transparent 1 ml syringes (Carl Roth, catalog number: H999.1)
  5. PVDF membrane (Merck Millipore, catalog number: IPVH00010)
  6. SDS-PAGE gel, 10-15% (w/v) polyacrylamide, prepare according to manufacturer’s instructions of the Mini-PROTEAN Tetra Cell SDS-PAGE system (Bio-Rad), store at 4 °C
  7. PageRuler prestained protein ladder (Thermo Fisher Scientific, catalog number: 26616)
  8. Avidin-HRP (Sigma-Aldrich, catalog number: A7419), store at -20 °C
  9. ECL reagent (Cell Signaling, catalog number: 6883), store at 4 °C
  10. Streptavidin agarose (Merck Millipore, catalog number: 69203), store at 4 °C
  11. BL21 Star (DE3)pLysS One Shot chemically competent *E. coli* (Thermo Fisher Scientific, catalog number: C602003), store at -80 °C
  12. Plasmid pHB111 (Beyer *et al.*, 2018b), store at -20 °C
  13. SEC column Superdex 200 Increase 10/300 GL (GE Healthcare, catalog number: 28990944), store at 4 °C
  14. Gel filtration standard (Bio-Rad, catalog number: 1511901), store at -20 °C
  15. Tris (Carl Roth, catalog number: AE15.2)
  16. Sodium dodecyl sulfate (SDS, Carl Roth, catalog number: CN30.3)
  17. 2-mercaptoethanol (Sigma-Aldrich, catalog number: M3148)
  18. Bromphenol blue sodium salt (Carl Roth, catalog number: A512.1)
  19. Zinc acetate dihydrate (Sigma-Aldrich, catalog number: 96459)
  20. Acetic acid (Carl Roth, catalog number: T179.2)
  21. 2-propanol (Carl Roth, catalog number: CP41.3)
  22. Coomassie brilliant blue R 250 (Carl Roth, catalog number: 3862.2)
  23. Ethanol (Carl Roth, catalog number: T171.3)
  24. Tween 20 (Carl Roth, catalog number: 9127.2)
  25. Ampicillin sodium salt (Carl Roth, catalog number: K029)
  26. Chloramphenicol (PanReac AppliChem, catalog number: A1806)
  27. 5x SDS loading buffer (see Recipes)
  28. Zinc solution (see Recipes)
  29. Coomassie solution (see Recipes)
  30. Destaining solution (see Recipes)
  31. Blocking buffer (see Recipes)
  32. Washing buffer (see Recipes)
  33. Beads buffer (see Recipes)
  34. 100 mg/ml ampicillin stock solution (see Recipes)
  35. 34 mg/ml chloramphenicol stock solution (see Recipes)
  36. Protein buffer (see Recipes)

## Equipment

- Production of PhyB-AviTag in *E. coli*
  1. 500 ml and 2 L baffled flasks, autoclave
  2. Thermoshaker (TSC ThermoShaker, Analytik Jena)
  3. Heated and refrigerated shaking incubator for bacteria (Innova 44R Shaker, orbit diameter: 2 in, Eppendorf)
  4. Photometer (GE Healthcare, model: GeneQuant 1300)
  5. Centrifuge Avanti J-26 XP (Beckman Coulter, model: Avanti J-26 XP) with JLA-8.1000 rotor (Beckman Coulter, model: JLA-8.1000) and 1 L centrifuge beakers (Beckman Coulter) for harvesting of bacteria
  6. Ultra low temperature freezer (-80 °C, Sanyo, model: MDF-U54V)
- Purification of PhyB-AviTag
  1. Water bath at 37 °C (PEQLAB, model: WB-4MS)
  2. High pressure homogenizer (SPX Flow Technology, model: APV-2000)
  3. Centrifuge Avanti J-26 XP (Beckman Coulter, model: Avanti J-26 XP) with JA-25.50 rotor (Beckman Coulter, model: JA-25.50) and 50 ml centrifuge beakers (Beckman Coulter) for clarification of lysate
  4. Fast protein liquid chromatography (FPLC) system (GE Healthcare, ÄKTAexplorer)
  5. Centrifuge Biofuge Primo R (Thermo Scientific, catalog number: 75005440) with rotor (Thermo Scientific, catalog number: 75007588) for centrifugal concentrators
  6. Centrifuge for 1.5 ml microcentrifuge tubes (Eppendorf, Centrifuge 5425 with rotor FA-24x2)
- Analysis of purified PhyB-AviTag
  1. Thermoshaker (TSC ThermoShaker, Analytik Jena)
  2. SDS-PAGE system (Bio-Rad, Mini-PROTEAN Tetra Cell)
  3. Ethidium bromide agarose gel imaging system (Gel iX Imager, Intas)
  4. Semi-dry electroblotting system (VWR, PerfectBlue)
  5. Chemiluminescence imaging system (GE Healthcare, ImageQuant LAS 4000 Mini)
  6. Centrifuge for 1.5 ml microcentrifuge tubes (Eppendorf, Centrifuge 5425 with rotor FA-24x2)
  7. Custom-build LED panels with 660 nm (LED660N-03) and 740 nm (LED740-01AU, both Roithner Lasertechnik) LEDs and adjustable intensity. For more information regarding the LED panels, see Müller *et al.* (2014)
  8. Fiber-optic spectrometer (Avantes, AvaSpec-ULS2048) to measure light intensities
  9. Green safe light (Osram, Deco Flex LED strips)
  10. Microplate reader (Tecan, model: Infinite M200 Pro)
  11. FPLC system (GE Healthcare, ÄKTAexplorer)

## Procedure

Note: If not stated otherwise, perform all steps at room temperature.

- Production of PhyB-AviTag in *E. coli*
  1. Transformation of *E. coli* BL21 Star (DE3) with plasmid pMH1105 encoding PhyB(1-651)-AviTag-His6 and the biosynthesis enzymes HO1 and PcyA:
    - Thaw 1 aliquot (50 µl) of *E. coli* BL21 Star (DE3) cells on ice.
    - Add 1 µl of pMH1105 plasmid DNA with a concentration of 10-1,000 ng/µl to the thawed bacteria and mix by gently tapping the tube.
    - Incubate on ice for 20 min.
    - Incubate in a Thermoshaker/heat block at 42 °C for 45 s.
    - Incubate on ice for 2 min.
    - Add 500 µl LB medium and incubate for 1 h in a Thermoshaker at 37 °C, 750 rpm.
    - Transfer bacteria to a 50 ml conical centrifuge tube containing 10 ml LB medium supplemented with 100 µg/ml streptomycin.
    - Incubate bacteria overnight (~15 h) at 37 °C, 150 rpm.
  2. On the next day, inoculate 100 ml of LB medium supplemented with 100 µg/ml streptomycin in a 500 ml baffled flask with the overnight culture (pre-culture).
  3. Incubate at 37 °C, 150 rpm for 4 h.
  4. Inoculate 6 flasks of 1 L LB medium supplemented with 100 µg/ml streptomycin in 2 L baffled flasks with each 10 ml of the pre-culture (expression cultures).
  5. Grow at 30 °C, 150 rpm until an OD_600_ of 0.6-0.8 is reached. Use cuvettes and a photometer to measure optical density at 600 nm using the same LB medium as blank.
  6. Induce protein production by adding 1 ml of 1 M IPTG stock solution to each expression culture (Final concentration = 1 mM).
  7. Add 10 ml of 5 mM biotin solution to each expression culture (Final concentration = 50 µM).
  8. Incubate at 18 °C, 150 rpm in the dark for 20 h (see Note 1).
  9. Harvest bacteria (expression culture should be slightly green, Figure 1A) by centrifugation at 6,500 *× g* for 10 min at 4 °C.
  10. Resuspend bacterial pellet (should be green, Figure 1B) in lysis buffer (30 ml lysis buffer per 1 L expression culture).
  11. Aliquot resuspended bacteria in 50 ml conical centrifuge tubes, shock freeze in liquid nitrogen and store at -80 °C.
    **Figure 1.**
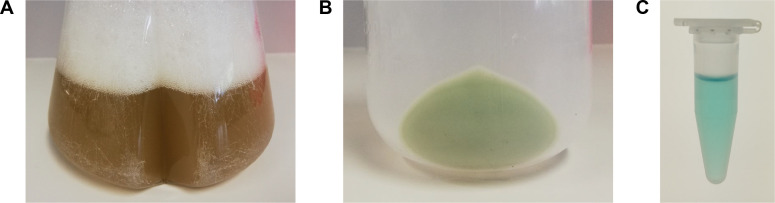
Pictures of A. the PhyB-AviTag-producing *E. coli* culture before harvesting, B. the corresponding bacterial pellet and C. the purified PhyB-AviTag protein. The culture had a green shading whereas the bacterial pellet was clearly green. Purified PhyB-AviTag (3 mg/ml) had a turquoise color.
- Purification of PhyB-AviTag
  1. Thaw tubes with the resuspended bacteria in a 37 °C water bath and immediately transfer them on ice once they are thawed.
  2. Lyse bacteria by 3 cycles in a high pressure homogenizer/French press at 1,000-1,500 bar (see Note 2).
  3. Clarify lysate by centrifugation at 30,000 *× g* for 30 min at 4 °C.
  4. Purify PhyB-AviTag from the supernatant by immobilized metal affinity chromatography (IMAC) using an FPLC/Äkta system at 4 °C (see Note 3):
    - Connect the Ni-NTA Superflow column to the system. For the purification process, use a flow rate of 5 ml/min. Monitor absorbance at 280 nm.
    - Load the lysate on the column. Load ~90 ml of lysate (equals ~3 L expression culture) per run.
    - Wash the column with 100 ml (equals 20 column volumes (CV)) lysis buffer.
    - Elute the protein with 50 ml (equals 10 CV) of elution buffer. Collect fractions (~2 ml) and merge the PhyB-AviTag containing turquoise colored fractions (Figure 1C).
  5. Optional: Concentrate the protein using a centrifugal concentrator at 8,000 *× g*. The buffer of up to 10 ml PhyB-AviTag can be exchanged in one desalting run (see next step).
  6. Exchange buffer of PhyB-AviTag to protein buffer using an FPLC/Äkta system:
    - Connect the HiPrep 26/10 desalting column to the system. For the buffer exchange, use a flow rate of 10 ml/min. Monitor absorbance at 280 nm and conductivity.
    - Equilibrate the column with 200 ml of protein buffer.
    - Load up to 10 ml of the (concentrated) IMAC eluate on the column.
    - Wash the column with 200 ml of protein buffer. Collect fractions (~4 ml) for the first 50 ml and merge the PhyB-AviTag containing turquoise colored fractions of the 280 nm absorbance peak (exclude fractions of the conductivity peak).
  7. Concentrate the protein using a centrifugal concentrator at 8,000 *× g* to a concentration of 1-5 mg/ml. Determine protein concentration by Bradford assay using BSA as standard (see Note 5).
  8. Optional: Freeze protein (see Note 6):
    - Add glycerol to a final concentration of 10% (v/v) to the protein.
    - Aliquot the protein solution in 1.5 ml microcentrifuge tubes, shock freeze in liquid nitrogen and store at -80 °C.
    - When needed, thaw the protein at 37 °C in a water bath or heat block. Once the protein is thawed, centrifuge protein at 20,000 *× g* for 5 min to pellet precipitates and store the supernatant at 4 °C.
- Analysis of purified PhyB-AviTag
  1. SDS-PAGE analysis of PhyB-AviTag (for representative results see Figure 2):
    - Mix 80 µl protein solution (~0.2 mg/ml) with 20 µl of 5x SDS loading buffer and incubate at 95 °C for 5 min in a Thermoshaker/heat block.
    - Separate protein samples by SDS-PAGE (10-15% (w/v) polyacrylamide gel) and continue with zinc and Coomassie staining (Step C1c) or detection of biotinylated protein (Step C1d).
    - Zinc and Coomassie staining:
      - Incubate the gel for 15 min in zinc solution and visualize zinc-induced fluorescence of the chromophore phycocyanobilin (PCB) upon illumination with UV light on a standard ethidium bromide agarose gel imaging system.
      - Incubate the gel for 1 h in Coomassie solution.
      - Incubate the gel overnight in destaining solution.
    - Detection of biotinylated protein:
      - Transfer proteins onto a PVDF membrane by electroblotting.
      - Block membrane by incubation for 1 h in blocking buffer.
      - Incubate membrane with Avidin-HRP diluted 1:2,000 in blocking buffer.
      - Wash membrane 3x for 5 min with washing buffer.
      - Visualize Avidin-HRP using ECL reagent and a chemiluminescence imaging system.
    **Figure 2.**
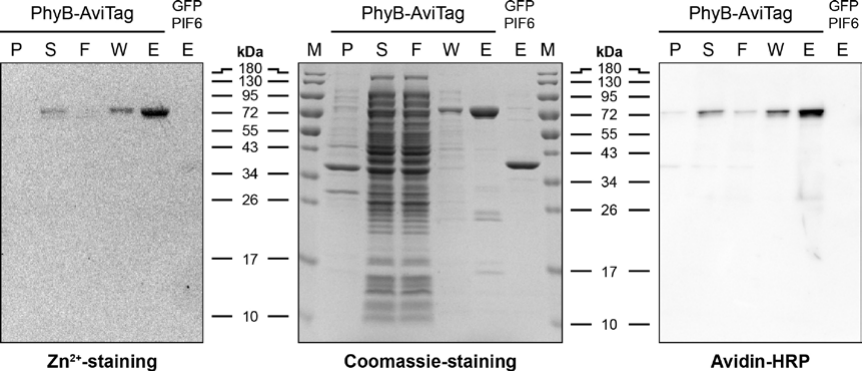
SDS-PAGE analysis of PhyB-AviTag and GFP-PIF6. The different fractions of the PhyB-AviTag purification process and purified GFP-PIF6 were separated by SDS-PAGE (15% (w/v) polyacrylamide) and stained for the chromophore phycocyanobilin (PCB, Zn^2+^-staining), proteins (Coomassie-staining) and biotin (Avidin-HRP). P, insoluble fraction of *E. coli* lysate; S, soluble fraction of lysate; F, flow-through of Ni-NTA column; W, wash of column; E, eluate of column. M, protein size marker. Expected PhyB-AviTag mass: 74 kDa. Expected GFP-PIF6 mass: 40 kDa.
  2. Estimation of biotinylation efficiency (for representative results see Note 7):
    - Transfer 400 µl of streptavidin agarose slurry (equivalent to 200 µl settled beads) into a 1.5 ml microcentrifuge tube and pellet beads by centrifugation at 500 *× g* for 3 min.
    - Wash beads by removing supernatant, resuspending beads in 1 ml beads buffer and pelleting beads again by centrifugation. Repeat this washing step 3 times.
    - Remove supernatant and resuspend beads in 200 µl beads buffer.
    - Dilute PhyB-AviTag in beads buffer to a concentration of ~80 µg/ml and make 200 µl aliquots into 6x 1.5 ml microcentrifuge tubes.
    - Add each 100 µl of beads suspension to 3 PhyB-AviTag containing tubes and each 100 µl of beads buffer to the 3 remaining tubes.
    - Incubate for 1 h while mixing tubes every 15 min.
    - Pellet beads by centrifugation and transfer 200 µl of the supernatants each into a new 1.5 ml microcentrifuge tube.
    - Pellet remaining beads by centrifugation and transfer 100 µl of the supernatants each into a well of a black 96-well plate. Transfer 100 µl of the PhyB-AviTag samples without beads each into a well of the same plate.
    - Illuminate proteins in the plate for 3 min with 740 nm light (intensity: 100 µmol/(m^2^·s)).
    - Transfer the plate in the dark (only dim green safe light) into the microplate reader and measure phytochrome fluorescence of the 6 samples (excitation: 635 nm, emission: 680 nm). Estimate the ratio of non-biotinylated PhyB-AviTag as the quotient of the fluorescence of the beads and the buffer samples.
  3. Spectral analysis of PhyB-AviTag photoswitching (for representative results see Figure 3):
    - Transfer 200 µl of PhyB-AviTag in protein buffer (concentration: ~1 mg/ml) into a well of a UV-transparent 96-well plate.
    - Illuminate protein for 3 min with 660 or 740 nm light (intensity: 100 µmol/(m^2^·s)).
    - Transfer the plate in the dark (only dim green safe light) into the microplate reader and measure absorbance spectra (250-900 nm, step size: 1 nm).
    - Repeat the measurement for the other illumination wavelength using the same sample.
    **Figure 3.**
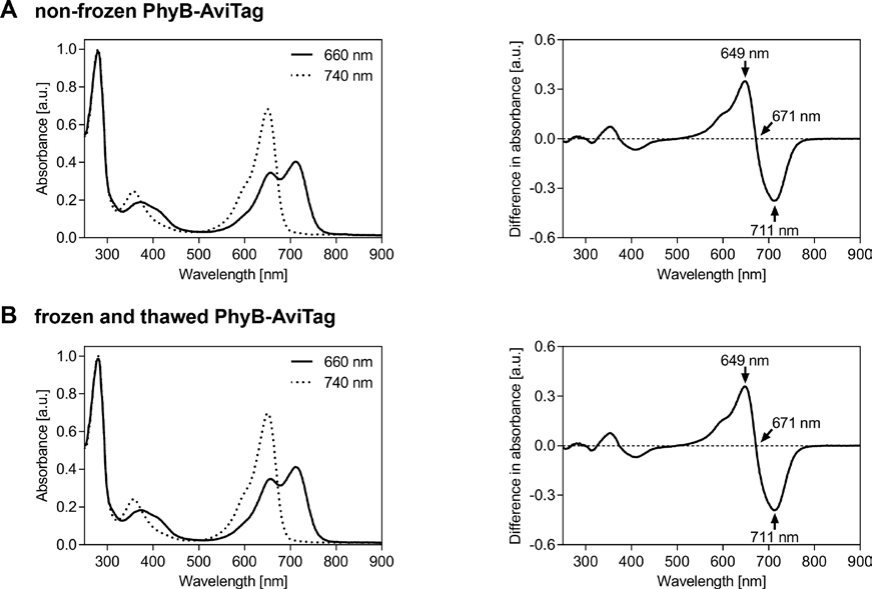
Spectral analysis of PhyB-AviTag photoswitching. PhyB-AviTag (1 mg/ml, never frozen A. and frozen and thawed B. was illuminated for 3 min with either 660 or 740 nm light (intensity: 100 µmol/(m^2^·s)) before acquisition of the absorbance spectra (left panels). The absorbance spectra in A. and B. were each normalized to 1 at 280 nm absorbance of the corresponding 740 nm spectrum to compensate for slight protein concentration variations. The difference spectra (right panels) were calculated by subtracting the 660 nm spectra from the corresponding 740 nm spectra. The peak wavelengths of the main peaks and of the isosbestic point are indicated. Freezing and thawing PhyB-AviTag once did not affect its absorbance spectra.
  4. Analysis of light-dependent PhyB-AviTag/PIF6 interaction by size exclusion chromatography (SEC, for representative results see Figure 4):
    - Produce and purify GFP-PIF6 as described for PhyB-AviTag with the following modifications:
      - Transform plasmid pHB111 (encoding GFP-PIF6(1-100)-His6 [Beyer *et al.*, 2018b]) into *E. coli* BL21 (DE3)pLysS and select for transformed cells with 100 µg/ml ampicillin and 34 µg/ml chloramphenicol.
      - After induction, incubate at 30 °C and 150 rpm for 6 h before harvesting.
    - Determine protein concentration of PhyB-AviTag and GFP-PIF6 by Bradford assay (see Procedure B).
    - Mix PhyB-AviTag (final concentration: ~2 mg/ml) with an equimolar amount of GFP-PIF6 in protein buffer (total volume: 800 µl) and distribute solution equally into 2 x 1.5 ml microcentrifuge tubes. For the molecular weights of both proteins, see legend of Figure 2.
    - SEC analysis:
      - Protect SEC column from light with aluminum foil and connect it to an FPLC/Äkta system equipped with a 200 µl sample loop.
      - Equilibrate column with 30 ml of protein buffer.
      - Load protein solution from one microcentrifuge tube into a transparent syringe and illuminate the protein for 3 min with 660 nm or 740 nm light (intensity: 100 µmol/(m^2^·s)).
      - Inject the protein in the dark (only dim green safe light) into the sample loop and start SEC run. Use a flow rate of 1 ml/min and monitor absorbance at 280 nm, 488 nm (GFP) and 671 nm (PhyB-AviTag). Once the protein is completely on the light-protected column (usually after a few ml), the room light can be switched on again.
      - Calibrate the column by performing one run with a gel filtration standard.
  **Figure 4.**
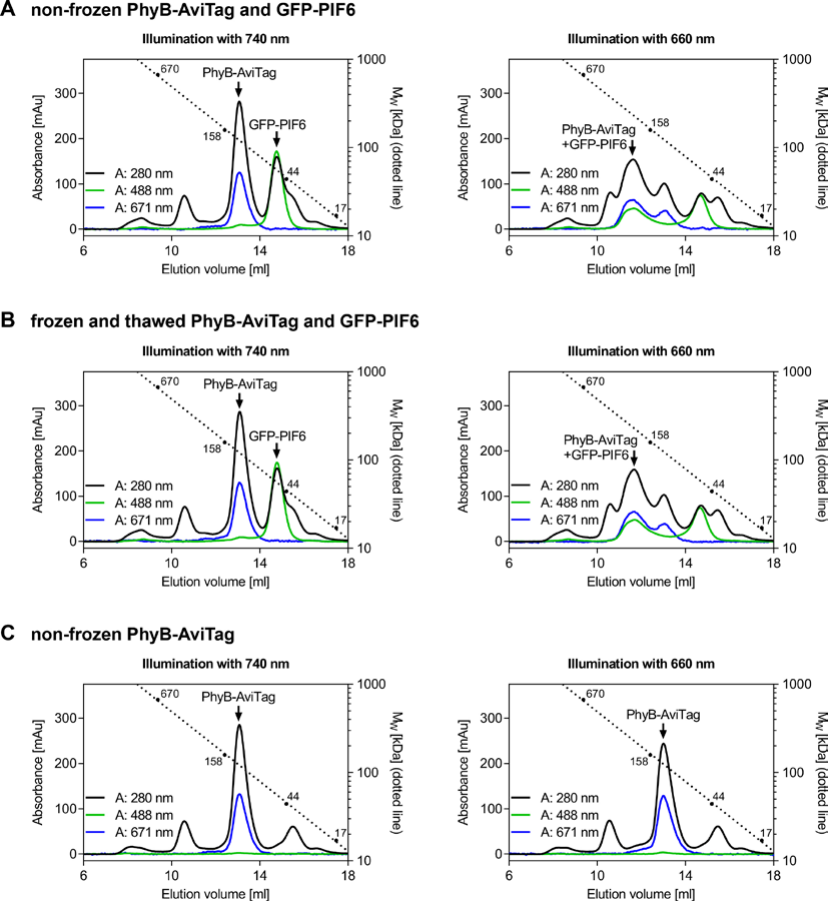
Analysis of light-dependent PhyB-AviTag/PIF6 interaction by size exclusion chromatography (SEC). A, B. 200 µl of an equimolar mixture of PhyB-AviTag (2.2 mg/ml) and GFP-PIF6 (1.2 mg/ml) was illuminated for 3 min with 660 or 740 nm light (intensity: 100 µmol/(m^2^·s)) and analyzed by SEC in the dark. Never frozen PhyB-AviTag and GFP-PIF6 A. and frozen and thawed proteins B. were analyzed. Freezing and thawing PhyB-AviTag and GFP-PIF6 once did not affect their light-dependent interaction. C. As control, 200 µl of never frozen PhyB-AviTag (2.2 mg/ml) was analyzed alone. The absorbance was monitored at 280 nm (total protein), 488 nm (absorption maximum of GFP-PIF6) and at 671 nm (isosbestic point of PhyB-AviTag). Calibration of the column with a gel filtration standard is indicated by the dotted line.

## Notes

1. Protect PhyB-AviTag (starting from induction of protein production in *E. coli*) from bright light such as direct sunlight. If possible, shade the lab, work under dim room light and protect PhyB-AviTag from light (*e.g.*, by covering with aluminum foil) whenever easily possible (*e.g.*, during incubation times). Working under green safe light (absorption minimum of PhyB-AviTag) is only required when the photostate of PhyB-AviTag should not be changed. Regular room light switches PhyB-AviTag usually similar as 660 nm light in the Pfr state, in which it interacts with PIF6.
2. As each passage of the sample through a high pressure homogenizer/French press heats the sample, cool the lysate on ice before the next passage. As alternative to a high pressure homogenizer/French press, bacteria can also be lysed by sonication.
3. Instead of using an FPLC/Äkta system for IMAC and buffer exchange, gravity flow columns can be used for small volumes/amounts.
4. The reducing agent TCEP is not particularly stable in phosphate buffers. Therefore, always add TCEP from the stock solution to PBS directly before use. Although TCEP is more stable in HEPES buffer, it is still recommended to always add TCEP directly before use.
5. Using the described purification protocol, the typical yield for PhyB-AviTag is ~4 mg of purified protein per 1 L expression culture. If high amounts of PhyB-AviTag are required, the protein can be produced in the multigram scale per 10 L high-cell-density fermentation run (Hörner *et al.*, 2019b).
6. PhyB-AviTag and GFP-PIF6 can be stored at 4 °C in the dark for at least two weeks. At -80 °C in the dark, the proteins can be stored for several years. Freeze the proteins in the presence of 10% (v/v) glycerol in aliquots and avoid repeated freeze-thaw cycles. Storing PhyB-AviTag and GFP-PIF6 at -80 °C overnight did not affect their light-dependent interaction and the spectra of PhyB-AviTag (Figures 3 and 4).
7. The estimation of the biotinylation efficiency described in Procedure, section C2 revealed ~80% biotinylation of PhyB-AviTag. If higher biotinylation is required, consider co-transformation of an additional plasmid encoding *E. coli* biotin ligase (BirA). Make sure to use a plasmid with a compatible origin of replication. The PhyB-AviTag plasmid pMH1105 is based on the commercial vector pCDFDuet (Novagen) with a CloDF13-derived CDF replicon (CDF ori) (Hörner *et al.*, 2019b).

## Recipes

Note: If not stated otherwise, store all solutions at room temperature. Use ultrapure water obtained by reverse osmosis for all solutions.

1. LB medium
  - Weigh 25 g LB and fill up to 1 L with water
  - Autoclave
2. 50 mg/ml streptomycin stock solution
  - Weigh 2.5 g streptomycin sulfate and fill up to 50 ml with water
  - Filter through a 0.22 µm PES syringe filter and store in aliquots at -20 °C
3. 1 M IPTG stock solution
  - Weigh 11.9 g IPTG and fill up to 50 ml with water
  - Filter through a 0.22 µm PES filter and store in aliquots at -20 °C
4. 5 mM biotin stock solution
  - Heat PBS to ~50 °C
  - Dissolve 84 mg biotin in 70 ml of heated PBS
  - Filter through a 0.22 µm PES filter. Always prepare fresh and cool down to < 37 °C before addition to bacterial culture
5. 100 mM TCEP stock solution, pH 8.0
  - Freshly prepare 0.5 M NaHCO_3_ in water, pH 9.0 (adjust with NaOH)
  - Weigh 2.87 g TCEP hydrochloride and fill up with 0.5 M NaHCO_3_ solution to 100 ml
  - Adjust pH to 8.0 with NaOH
  - Store in single-use aliquots at -20 °C
6. Lysis buffer, pH 8.0 (adjust with NaOH)
  100 mM HEPES
  500 mM NaCl
  20 mM imidazole
  5% (v/v) glycerol
  0.5 mM TCEP [add from TCEP stock solution (see Recipe 5) directly before use (see Note 4)]
  Store at 4 °C (before addition of TCEP)
7. Elution buffer, pH 8.0 (adjust with HCl)
  50 mM HEPES
  500 mM NaCl
  500 mM imidazole
  5% (v/v) glycerol
  0.5 mM TCEP [add from TCEP stock solution (see Recipe 5) directly before use (see Note 4)]
  Store at 4 °C (before addition of TCEP)
8. Protein buffer
  PBS
  0.5 mM TCEP [add from TCEP stock solution (see Recipe 5) directly before use (see Note 4)]
9. 1 mg/ml BSA protein standard
  - Weigh 500 mg BSA and fill up to 500 ml with water
  - Store in single-use aliquots at -20 °C
10. 5x SDS loading buffer
  0.31 M Tris-HCl (from 1 M Tris-HCl stock solution, pH 6.8)
  50% (v/v) glycerol
  10% (w/v) sodium dodecyl sulfate
  12.5% (v/v) 2-mercaptoethanol
  0.05% (w/v) bromphenol blue sodium salt
  Store in single-use aliquots at -20 °C
11. Zinc solution
  1 mM zinc acetate dihydrate
12. Coomassie solution
  10% (v/v) acetic acid
  25% (v/v) 2-propanol
  0.1% (w/v) Coomassie brilliant blue R 250
13. Destaining solution
  10% (v/v) acetic acid
  20% (v/v) ethanol
14. Blocking buffer
  PBS with 3% (w/v) BSA
15. Washing buffer
  PBS with 0.05% (v/v) Tween 20
16. Beads buffer
  PBS with 1% (w/v) BSA
17. 100 mg/ml ampicillin stock solution
  - Weigh 5 g ampicillin sodium salt and fill up to 50 ml with water
  - Filter through a 0.22 µm PES filter and store in aliquots at -20 °C
18. 34 mg/ml chloramphenicol stock solution
  - Weigh 1.7 g chloramphenicol and fill up to 50 ml with ethanol
  - Store in aliquots at -20 °C
