# Supplementary material for: Production, Purification and Characterization of Recombinant Biotinylated Phytochrome B for Extracellular Optogenetics
Source: Bio Protoc. 2020 Mar 5;10(5):e3541. doi: 10.21769/BioProtoc.3541 (PMC7842835; doi:10.21769/BioProtoc.3541)
Supplement: Table S1 [file BioProtoc-10-05-3541-s001.docx]

**Supplementary Information**

**Table S1. Nucleic acid sequence of plasmid pMH1105 described in this protocol.** The plasmid is based on our previously published plasmid pMH610 (Hörner *et al.*, 2019b) and uses the commercial vector pCDFDuet-1 (Novagen) as backbone. The complete annotated plasmid sequence can be found on Addgene, plasmid ID 131864.

| **Plasmid** | **Nucleic acid sequence (5′ 🡪 3′)** |
| --- | --- |
| **pMH1105**  T7 promoter  PhyB (1-651)  AviTag  His6  HO1  PcyA  Backbone:  pCDFDuet-1 | ···ATTAGGAAATTAATACGACTCACTATAGGGGAATTGTGAGCGGATAACAATTCCCCTGTAGAAATAATTTTGTTTAACTTTAATAAGGAGATATACCATGGTTAGCGGTGTTGGTGGTAGCGGTGGTGGTCGTGGTGGCGGTCGCGGAGGTGAAGAAGAACCGAGCAGCAGCCATACCCCGAATAATCGCCGTGGTGGTGAACAGGCACAGAGCAGCGGCACCAAAAGCCTGCGTCCGCGTAGCAATACCGAAAGCATGAGCAAAGCAATTCAGCAGTATACCGTTGATGCACGTCTGCATGCCGTTTTCGAACAGAGCGGTGAAAGCGGTAAAAGCTTTGATTATAGCCAGAGCCTGAAAACCACCACCTATGGTAGCAGCGTGCCGGAACAGCAGATTACCGCATATCTGAGCCGTATTCAGCGTGGTGGCTACATTCAGCCGTTTGGCTGCATGATCGCAGTTGATGAAAGCAGCTTTCGCATTATTGGCTACAGCGAAAATGCACGTGAAATGCTGGGCATTATGCCGCAGAGCGTTCCGACCCTGGAAAAACCGGAAATTCTGGCAATGGGCACCGATGTTCGTAGCCTGTTTACCAGCAGCAGCTCCATTCTGCTGGAACGTGCCTTTGTTGCCCGTGAAATTACCCTGCTGAATCCGGTTTGGATTCATAGCAAAAACACCGGCAAACCGTTTTATGCAATTCTGCATCGTATTGATGTTGGCGTGGTTATTGATCTGGAACCGGCACGTACCGAAGATCCGGCACTGAGCATTGCCGGTGCAGTTCAGAGCCAGAAACTGGCAGTTCGTGCAATTAGCCAGCTGCAGGCACTGCCTGGTGGTGATATCAAACTGCTGTGTGATACCGTTGTTGAAAGCGTTCGTGATCTGACCGGCTACGACCGTGTTATGGTGTATAAATTCCACGAAGATGAACATGGTGAAGTTGTTGCAGAAAGCAAACGTGATGACCTGGAACCGTATATTGGTCTGCATTATCCAGCAACCGATATTCCGCAGGCAAGCCGTTTCCTGTTCAAACAGAATCGTGTGCGCATGATTGTTGATTGTAATGCAACACCGGTTCTGGTTGTTCAGGATGATCGTCTGACCCAGAGCATGTGTCTGGTTGGTAGCACCCTGCGTGCACCGCATGGTTGTCATAGCCAGTATATGGCAAATATGGGTAGCATCGCAAGCCTGGCCATGGCGGTGATCATCAATGGTAATGAAGATGATGGTAGCAATGTTGCAAGCGGTCGTAGCAGCATGCGTCTGTGGGGTCTGGTTGTGTGTCATCATACCAGCAGTCGCTGCATTCCGTTTCCGCTGCGTTATGCATGTGAATTTCTGATGCAGGCATTTGGACTGCAGCTGAATATGGAACTGCAACTGGCACTGCAGATGAGCGAAAAACGTGTTCTGCGTACCCAGACCCTGCTGTGCGATATGCTGCTGCGTGATAGTCCGGCAGGCATTGTTACCCAGAGCCCGAGCATTATGGATCTGGTGAAATGCGATGGTGCAGCCTTTCTGTATCACGGTAAATACTATCCGCTGGGTGTTGCACCGAGCGAAGTTCAGATTAAAGATGTTGTTGAGTGGCTGCTGGCAAATCATGCAGATAGCACCGGTCTGAGCACCGATAGCCTGGGTGATGCAGGTTATCCGGGTGCAGCAGCACTGGGAGATGCAGTTTGTGGTATGGCAGTTGCATACATTACCAAACGCGATTTTCTGTTTTGGTTTCGTAGCCATACCGCCAAAGAAATCAAATGGGGTGGTGCAAAACATCACCCGGAAGATAAAGATGACGGTCAGCGTATGCATCCGCGTAGTAGCTTTCAGGCATTTCTGGAAGTGGTGAAAAGCCGTAGCCAGCCGTGGGAAACCGCAGAAATGGATGCAATTCATAGCCTGCAACTGATTCTGCGCGATAGCTTCAAAGAAAGCGAAGCAGCAATGAATAGCAAAGTTGTTGATGGTGTTGTTCAGCCGTGTCGTGATATGGCAGGCGAACAGGGTATTGATGAACTGGGTGCAGGTTCTGGTAGCGGTCTGAACGACATCTTCGAAGCTCAGAAAATCGAATGGCACGAACATCATCACCATCACCATTAAGCGGCCGCATAATGCTTAAGTCGAACAGAAAGTAATCGTATTGTACACGGCCGCATAATCGAAATTAATACGACTCACTATAGGGGAATTGTGAGCGGATAACAATTCCCCATCTTAGTATATTAGTTAAGTATAAGAAGGAGATATACATATGAGTGTCAACTTAGCTTCCCAGTTGCGGGAAGGGACGAAAAAATCCCACTCCATGGCGGAGAACGTCGGCTTTGTCAAATGCTTCCTCAAGGGCGTTGTCGAGAAAAATTCCTACCGTAAGCTGGTTGGCAATCTCTACTTTGTCTACAGTGCCATGGAAGAGGAAATGGCAAAATTTAAGGACCATCCCATCCTCAGCCACATTTACTTCCCCGAACTCAACCGCAAACAAAGCCTAGAGCAAGACCTGCAATTCTATTACGGCTCCAACTGGCGGCAAGAAGTGAAAATTTCTGCCGCTGGCCAAGCCTATGTGGACCGAGTCCGGCAAGTGGCCGCTACGGCCCCTGAATTGTTGGTGGCCCATTCCTACACCCGTTACCTGGGGGATCTTTCCGGCGGTCAAATTCTCAAGAAAATTGCCCAAAATGCCATGAATCTCCACGATGGTGGCACAGCTTTCTATGAATTTGCCGACATTGATGACGAAAAGGCTTTTAAAAATACCTACCGTCAAGCTATGAATGATCTGCCCATTGACCAAGCCACCGCCGAACGGATTGTGGATGAAGCCAATGACGCCTTTGCCATGAACATGAAAATGTTCAACGAACTTGAAGGCAACCTGATCAAGGCGATCGGCATTATGGTGTTCAACAGCCTCACCCGTCGCCGCAGTCAAGGCAGCACCGAAGTTGGCCTCGCCACCTCCGAAGGCTAGAAGCTTGGATCCATTAAAGAGGAGAAACCCGGGCATGGCCGTCACTGATTTAAGTTTGACCAATTCTTCCCTGATGCCTACGTTGAACCCGATGATTCAACAGTTGGCCCTGGCGATCGCCGCTAGTTGGCAAAGTTTACCCCTCAAGCCCTATCAATTGCCGGAGGATTTGGGCTACGTAGAAGGCCGCCTGGAAGGGGAAAAGTTAGTGATTGAAAATCGGTGCTACCAAACGCCCCAGTTTCGCAAAATGCATTTGGAGTTGGCCAAGGTGGGCAAAGGGTTGGATATTCTCCACTGTGTAATGTTTCCTGAGCCTTTATACGGTCTACCTTTGTTTGGCTGTGACATTGTGGCCGGCCCCGGTGGAGTAAGTGCGGCTATTGCGGATCTATCCCCCACCCAAAGCGATCGCCAATTGCCCGCAGCGTACCAAAAATCATTGGCAGAGCTAGGCCAGCCAGAATTTGAGCAACAACGGGAATTGCCCCCCTGGGGAGAAATATTTTCTGAATATTGTTTATTCATCCGTCCCAGCAATGTCACTGAAGAAGAAAGATTTGTACAAAGGGTAGTGGACTTTTTGCAAATTCATTGTCACCAATCCATCGTTGCCGAACCCTTGTCTGAAGCTCAAACTTTGGAGCACCGTCAGGGGCAAATTCATTACTGCCAACAACAACAGAAAAATGATAAAACCCGTCGGGTACTGGAAAAAGCTTTTGGGGAAGCTTGGGCGGAACGGTATATGAGCCAAGTCTTATTTGATGTTATCCAATAAGACGTCGGTACCCTCGAGTCTGGTAAAGAAACCGCTGCTGCGAAATTGAACGCCA··· |
